# Etiological composition and epidemiological characteristics of acute respiratory infections in Hebei Province, 2023–2025

**DOI:** 10.3389/fpubh.2026.1883970

**Published:** 2026-08-14

**Authors:** Nana Guo, Ziyi Pang, Yan Li, Caixiao Jiang, Minghao Geng, Wentao Wu, Guangyue Han, Xu Han, Qi Li

**Affiliations:** 1 Hebei Provincial Center for Disease Control and Prevention, Shijiazhuang, Hebei, China; 2 School of Public Health, Hebei University, Baoding, Hebei, China

**Keywords:** coinfection, epidemiology, Hebei Province, pathogen spectrum, respiratory tract infections

## Abstract

**Background:**

Acute respiratory tract infections (ARTIs) are a major global public health concern. This study aimed to characterize the pathogen spectrum and epidemiological features of 11 common respiratory pathogens among outpatients and hospitalized patients in Hebei Province.

**Methods:**

A total of 11,942 patients with influenza-like illness (ILI) or severe acute respiratory infection (SARI) were enrolled from October 2023 to September 2025. Throat swabs, sputum, and bronchoalveolar lavage fluid were collected and screened using a multiplex PCR kit. Age distribution, temporal trends, and coinfection patterns were analyzed.

**Results:**

The results showed that the pathogen positivity rate among SARI (45.71%) was higher than that among ILI (41.62%); influenza virus (IFV) had the highest detection rate among ILI, whilst Mycoplasma pneumoniae (M. pneumoniae) was the most frequently detected pathogen among SARI; In terms of age distribution: IFV had the highest detection rate across all age groups in ILI; in the 0–4-year-old SARI group, respiratory syncytial virus was predominant; in the 5–14-year-old group, the detection rate of M. pneumoniae reached 43.44 %; and in the group aged 15 years and over, IFV and Severe Acute Respiratory Syndrome Coronavirus 2(SARS-CoV-2) were the predominant pathogens. SARS-CoV-2 were intermittently detected throughout the year, with three waves of epidemic peaks observed. IFV and RSV predominantly emerged during winter and spring, while rhinovirus showed higher activity in summer and autumn. M. pneumoniae exhibited a high incidence rate initially, which has been declining steadily since the end of 2024.The proportion of multi-pathogen co-infections among positive specimens was 14.88%, the proportion of co-infections among SARI (23.76 %) was significantly higher than that among ILI (9.28 %). Co-detection of M. pneumoniae and rhinovirus was the most common, followed by human bocavirus, parainfluenza virus and enterovirus, which were also among the pathogens frequently detected in combination.

**Conclusion:**

This study elucidated the epidemiological characteristics of respiratory pathogens in Hebei Province, providing a scientific basis for the precise prevention and control of ARTIs.

## 1 Introduction

Acute respiratory tract infections (ARTIs) are a globally prevalent group of infectious diseases caused by diverse pathogenic microorganisms, including bacteria, viruses, and mycoplasmas. Characterized by rapid transmission, wide reach, and high population susceptibility, ARIs pose a severe threat to human health and represent a major public health concern (1, 2). According to surveillance data from the World Health Organization (WHO) in 2021, ARTIs remain a leading cause of mortality worldwide, imposing a substantial burden on healthcare systems and socioeconomic development.

In China, ARTIs are consistently among the primary reasons for outpatient and hospital admissions (3). They frequently lead to clustered outbreaks, particularly during winter and spring, posing a continuous threat to the health of various age groups (4, 5). In recent years, the alternating circulation and mutation of pathogens such as SARS-CoV-2 and influenza viruses have resulted in an increasingly complex and diverse etiological profile of respiratory infections. Significant variations in epidemiological patterns, pathogenicity, and susceptible populations among different pathogens present substantial challenges to clinical diagnosis, treatment, and precise prevention and control (6). For instance, the seasonal outbreaks of influenza and the emergence of multiple-pathogen coinfections further complicate epidemic control, necessitating systematic surveillance to clarify regional epidemiological characteristics (7).

Therefore, conducting pathogen surveillance among influenza-like illness (ILI) cases in sentinel hospital outpatient departments and severe acute respiratory infection (SARI) inpatients is crucial for understanding the composition of the regional respiratory pathogen spectrum, epidemiological trends, age distribution, and coinfection status. Hebei Province, China, has conducted active surveillance for ARIs since 2009, initially targeting common pathogens such as influenza, measles, mumps, pertussis, tuberculosis, and scarlet fever. However, the epidemiological characteristics of acute respiratory pathogens have shifted significantly following the COVID-19 pandemic. Furthermore, with advancements in detection technology, the scope of pathogen surveillance in Hebei Province was expanded in 2023. Utilizing these updated data, we aimed to determine the etiological and epidemiological characteristics of ARTIs across all age groups in Hebei Province.

Previous studies have lacked a systematic analysis of long-term, multi-pathogen surveillance data in Hebei Province, limiting the comprehensive support for precise prevention and control strategies. Consequently, based on surveillance data from October 2023 to September 2025, this study systematically analyzed the epidemiological characteristics of respiratory pathogens in Hebei Province during this period. These findings provide a scientific basis for optimizing prevention and control strategies, rationally allocating medical resources, and improving clinical diagnosis and treatment efficiency, which is of significant practical importance for reducing the morbidity and mortality associated with respiratory infections.

## 2 Methods

### 2.1 Study population

The study selected 11 sentinel hospitals in Hebei Province. These 11 hospitals—which include children's hospitals, general hospitals, infectious disease hospitals and hospitals practicing a combination of traditional Chinese and Western medicine—are spread across 11 prefecture-level cities in Hebei Province. All sentinel hospitals are Grade III Class A or Grade III specialist hospitals in their respective regions, and are equipped with well-established facilities for the diagnosis and treatment of respiratory diseases and for pathogen detection. This study collected a total of 11,942 samples, including influenza-like illness (ILI) samples from outpatients and severe acute respiratory infection (SARI) samples from inpatients, who were admitted to Sentinel hospitals for acute respiratory tract infections (ARTIs) surveillance in Hebei Province during the period from October 2023 to September 2025. ILI cases were defined according to the National Influenza Surveillance Program as patients with acute onset, fever (temperature ≥38 °C), accompanied by either cough or sore throat, and a disease duration of ≤ 3 days. SARI cases were defined based on the Guidelines for the Diagnosis and Treatment of Acute Respiratory Infections as patients requiring hospitalization who presented with at least one respiratory symptom (including cough, sore throat, nasal congestion, rhinorrhea, or fever ≥37.3 °C) within a duration of ≤ 14 days, and were clinically diagnosed with acute upper respiratory infection, acute lower respiratory infection, acute bronchitis, or pneumonia.

This study was conducted in strict accordance with the ethical principles of the Declaration of Helsinki and was approved by the Ethics Committee of the Hebei Provincial Center for Disease Control and Prevention (Approval No.: HeBIRBS2024020). Given that this is a retrospective observational study, the respiratory specimens used were all samples collected during routine surveillance of acute respiratory infections in Hebei Province, with no identifiable personal information of the participants collected. The Ethics Committee officially granted a waiver of informed consent for this study in accordance with the relevant national ethical guidelines and the provisions of the Declaration of Helsinki. All research procedures were strictly compliant with the ethical norms for public health surveillance studies and the relevant regulations on biosafety management.

### 2.2 Specimen collection

Specimens were collected by trained professional medical personnel from respiratory multi-pathogen surveillance sentinel hospitals. For ILI cases, cases within 3 days of onset were collected; sampling types included throat swabs, nasal swabs, or nasopharyngeal swabs and other respiratory samples.

From April to September, each sentinel hospital collects 20 eligible samples per month; from October to March of the following year, an average of 20 samples are collected each week to ensure a balanced distribution of cases across age groups and time periods. For SARI cases, cases within 1 week of onset were preferentially collected (if the number of cases cannot meet the requirements, it can be relaxed to within 10 days of onset); sampling types included deep sputum, bronchoalveolar lavage fluid, throat swabs, nasal swabs, or nasopharyngeal swabs and other respiratory samples. Deep sputum, bronchoalveolar lavage fluid and other lower respiratory tract samples were preferentially collected. It is required to collect 2 tubes of the same type of sample (1 tube with antibiotics added, 1 tube without antibiotics added). From April to September, each sentinel hospital collected 10 compliant samples per month; from October to March of the following year, an average of 10 samples were collected weekly. This ensured balanced distribution of onset times and age groups among the sampled individuals. All specimens must be placed in screw-cap sampling tubes fitted with gaskets; the outer surface must be labeled with the sample number, date of collection, case classification and specimen type, and the tubes must be double-sealed before dispatch. Following collection, specimens must be stored at room temperature for no longer than 4 h and transported via the cold chain at 2–8 °C, ensuring delivery to the laboratory within 3 h of collection; Specimens that cannot be tested immediately should be stored at 4 °C for a short period ( ≤ 24 h); if storage exceeds 24 h, they should be aliquoted and stored in a freezer at −80 °C, taking care to avoid repeated freeze-thaw cycles throughout the process. Upon receipt at the laboratory, specimens must be signed for by two members of staff, who shall check for any leakage or damage; non-compliant specimens shall be returned for re-collection.

### 2.3 Multi-pathogen detection

According to the corresponding requirements and operation procedures of the DNA/RNA Extraction Kit (Nucleic Acid Extraction Kit from Shanghai Biogerm Medical Technology Co., Ltd.), nucleic acid was extracted from throat swabs. The elution volume should not be less than 50 μL. Subsequently, the National Acute Respiratory Infectious Disease Multiple Pathogen Rapid Detection Kit (Version A, TaqMan probe method, Beijing Zhuocheng Huisheng Biotechnology Co., Ltd.) was used for multi-pathogen detection. The kit has a lower limit of detection of 5 × 10^2^ copies/ml, a linear range of 5 × 10^2^ to 2 × 10^10^ copies/ml, and a precision (CV) of < 5%; the kit meets the requirements of the experiment. Positive results were interpreted according to the kit instructions, i.e., when the cycle threshold (Ct) > 38 or not detected, it was judged as negative. When the amplification curve was S-shaped and the Ct value ≤ 38, it was judged as positive. The detected respiratory pathogens included: Severe Acute Respiratory Syndrome Coronavirus 2 (SARS-CoV-2), influenza virus (IFV), respiratory syncytial virus (RSV), adenovirus (HAdV), human metapneumovirus (HMPV), human parainfluenza viruses (HPIV), Human Coronavirus (HCoV), human bocavirus (HBoV), human rhinovirus (HRV), enterovirus (HEV), and Mycoplasma pneumoniae (M. pneumoniae).

### 2.4 Statistical analysis

Data collation was performed using Excel software, and statistical analysis was performed using R 4.5.1 software. Case numbers and percentages were used for statistical description of baseline characteristics. ILI cases and SARI cases were respectively subjected to subgroup analysis according to age, time, and mixed infection status. Comparison of differences between groups was performed using the chi-square test or Fisher's exact probability method. A *P*-value < 0.05 was considered statistically significant.

## 3 Results

### 3.1 Respiratory pathogen detection results

Between October 2023 and October 2025 (a total of 103 weeks), a total of 11,942 specimens were collected, comprising 7,587 from ILI and 4,355 from SARI. During the surveillance period, 11 types of respiratory pathogens were detected across the entire population. Of the 11,942 specimens, 5,149 tested positive, yielding an overall positivity rate of 43.12 %. Among these, the pathogen with the highest detection rate was IFV (9.65%), followed by M. pneumoniae (8.74%) and HRV (6.28%), whilst HCoV (2.16%), HEV (1.83%) and HBoV (1.28%) generally had lower detection rates among respiratory pathogens in Hebei Province.

**Figure 1:**
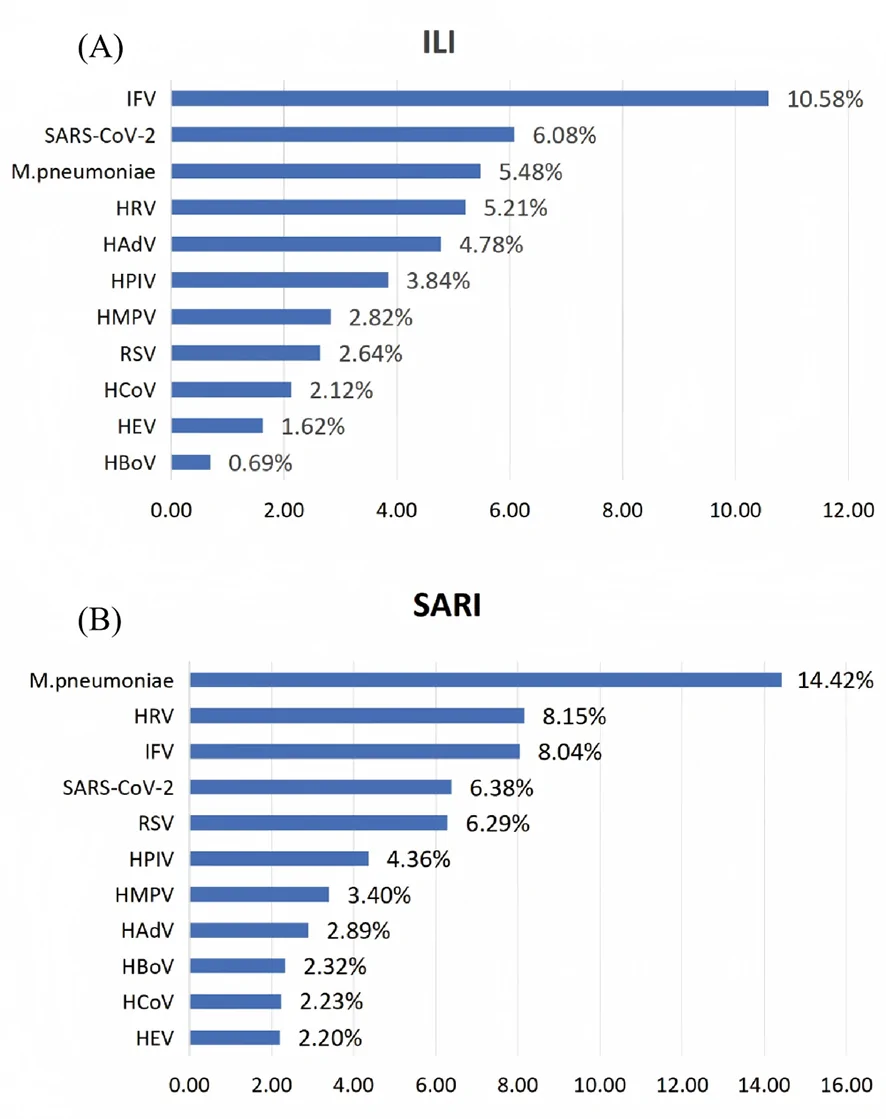
Pathogen detection status in ILI and SARI: **(A)** Summary of pathogen detection rates in ILI from October 2023 to September 2025; **(B)** Summary of pathogen detection rates in SARI from October 2023 to September 2025.

Among ILI patients, 41.62% (3,158/7,587) tested positive for at least one pathogen; amongst SARI, 1,991 cases tested positive for at least one pathogen, with a positivity rate of 45.71%. The difference between ILI and SARI was statistically significant (χ^2^ = 18.906, *P* < 0.001). Among ILI, the detection rate for IFV (10.58%) remained the highest, followed by SARS-CoV-2 (6.08%) and M. pneumoniae (5.48%); among SARI, the pathogens with higher detection rates were M. pneumoniae (14.42%), HRV (8.15%) and IFV (8.04%); see Figure 1 for further details.

A chi-squared test was further employed to compare the differences in the positivity rates for various pathogens between the ILI and SARI groups; the statistical results are shown in Table 1. There was no statistically significant difference in the detection rates of the four pathogens—SARS-CoV-2, HMPV, HPIV and HCoV—between the ILI and SARI groups (*P* > 0.05). Statistically significant differences were observed in the positivity rates for the remaining pathogens between the two groups (*P* < 0.05). Specifically, the positivity rates for IFV and HAdV were significantly higher in the ILI group than in the SARI group;whilst the positivity rates for RSV, HBoV, HRV, HEV and M. pneumoniae were significantly higher in the SARI group than in the ILI group.

**Table 1:** Comparison of pathogen positive rates between ILI and SARI groups using chi-square test.

Pathogen species	ILI (*N* =7,587)		SARI (*N* = 4,355)		*X* ^2^	*P*
	Positive cases (*n*)	Detection rate (%)	Positive cases (*n*)	Detection rate (%)		
SARS-CoV-2	461	6.08	278	6.38	0.39865	0.5278
IFV	803	10.58	350	8.04	20.289	**< 0.0001**
RSV	200	2.64	274	6.29	96.043	**< 0.0001**
HAdV	363	4.78	126	2.89	24.722	**< 0.0001**
HMPV	214	2.82	142	3.40	1.7031	0.1919
HPIV	291	3.84	190	4.36	1.856	0.1731
HCoV	161	2.12	97	2.23	0.099537	0.7524
HBoV	52	0.69	101	2.32	57.108	**< 0.0001**
HRV	395	5.21	355	8.15	40.278	**< 0.0001**
HEV	123	1.62	96	2.20	4.9079	**0.02673**
M. pneumoniae	416	5.48	628	14.42	275.88	**< 0.0001**

Values in bold indicate statistically significant differences (*P* < 0.05).

This study categorized the detection of pathogens in different types of respiratory specimens according to ILI and SARI groups. The vast majority of ILI cases were tested using throat swabs; the overall pathogen detection rate for this type of specimen was 41.73% (3,158/7,567). The composition of specimens from SARI cases differed markedly: throat swabs accounted for 43.44% (1,892/4,355) of all SARI specimens, with a corresponding pathogen detection rate of 43.45% (822/1,892); lower respiratory tract samples, such as sputum and bronchoalveolar lavage fluid, accounted for 47.46% (2,463/4,355) of the total SARI samples, with a pathogen detection rate of 47.46% (1,169/2,463). The results of the between-group chi-square test showed that there was no statistically significant difference in pathogen detection rates between ILI throat swabs and SARI throat swabs (χ^2^ = 1.7744, *P* = 0.1828); however, within the SARI cohort, there was a statistically significant difference in pathogen detection rates between throat swabs and lower respiratory tract samples (χ^2^ = 6.7945, *P* = 0.009144), with lower respiratory tract samples demonstrating higher detection efficiency.

### 3.2 Age-specific pathogen detection

The patients included in the study were divided into four age groups: 0–4 years (*n* = 3,323), 5–14 years (*n* = 2,751), 15–59 years (*n* = 3,308) and >60 years (*n* = 2,560). Overall, the detection rates of respiratory pathogens across the different age groups were, in descending order: 0–4 years (55.16 %, 1,833/3,323), 5–14 years (56.56%, 1,556/2,751), 15–59 years (30.86%, 1,012/3,308) and >60 years (29.22%, 748/2,560). The differences in overall detection rates between the different age groups were statistically significant (χ^2^ = 814.26, *P* < 0.01).

**Figure 2:**
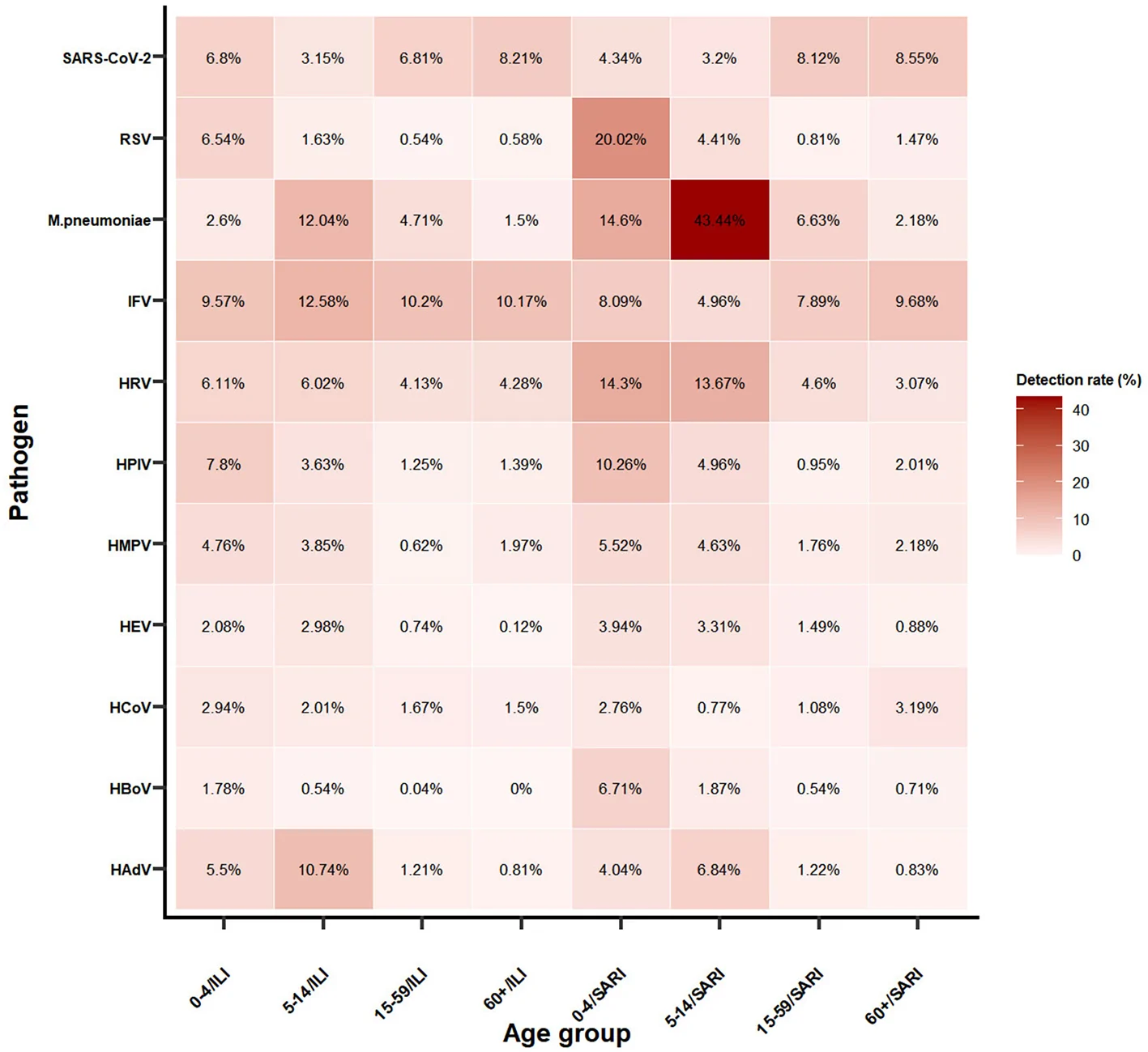
Distribution of pathogen detection rates in ILI cases and SARI cases across different age groups.

Among ILI cases, the 5–14 age group had the highest overall detection rate of respiratory pathogens, at 51.79%, followed by the 0–4 age group at 50.97%. There was marked heterogeneity in the composition of predominant pathogens across different age groups: in the 0–4-year-old group, IFV was the main pathogen detected, with a positivity rate of 9.57%; IFV was most prevalent in the 5–14-year-old age group, with a detection rate of 12.58%; the 15–59-year-old group also had the highest IFV positivity rate (10.20%); in the over-60 age group, IFV (10.17%) and SARS-CoV-2 (8.21%) were the main pathogens detected. The specific pathogen detection patterns across different age groups in outpatient and emergency department cases are shown in Figure 2 and Table 2.

**Table 2:** Detection of pathogens by age group among ILI cases.

Pathogen species	ILI				*P*
	0–4 years (*N* = 2,309) *n* (%)	5–14 years (*N* = 1,844) *n* (%)	15–59 years (*N* = 2,569) *n* (%)	>60 years (*N* = 865) *n* (%)	
SARS-CoV-2	157 (6.80%)	58 (3.15%)	175 (6.81%)	71 (8.21%)	**< 0.001**
IFV	221 (9.57%)	232 (12.58%)	262 (10.20%)	88 (10.17%)	**0.013**
RSV	151 (6.54%)	30 (1.63%)	14 (0.54%)	5 (0.58%)	**< 0.001**
HAdV	127 (5.50%)	198 (10.74%)	31 (1.21%)	7 (0.81%)	**< 0.001**
HMPV	110 (4.76%)	71 (3.85%)	16 (0.62%)	17 (1.97%)	**< 0.001**
HPIV	180 (7.80%)	67 (3.63%)	32 (1.25%)	12 (1.39%)	**< 0.001**
HCoV	68 (2.94%)	37 (2.01%)	43 (1.67%)	13 (1.50%)	**0.008**
HBoV	41 (1.78%)	10 (0.54%)	1 (0.04%)	0 (0.00%)	**< 0.001**
HRV	141 (6.11%)	111 (6.02%)	106 (4.13%)	37 (4.28%)	**0.003**
HEV	48 (2.08%)	55 (2.98%)	19 (0.74%)	1 (0.12%)	**< 0.001**
M.pneumoniae	60 (2.60%)	222 (12.04%)	121 (4.71%)	13 (1.50%)	**< 0.001**

Values in bold indicate statistically significant differences (*P* < 0.05).

Among SARI cases, the 5–14 age group had the highest overall positive detection rate for respiratory pathogens (66.26%), followed by the 0–4 age group (64.69%). Furthermore, there were significant differences in the composition of predominant pathogens across age groups: RSV (20.02%) was the predominant pathogen in the 0–4 age group, whilst M. pneumoniae (43.44%) had the highest detection rate in the 5–14 age group, whilst in the 15–59-year-old and over-60 age groups, SARS-CoV-2 (8.12%and 8.55 %) and IFV (7.98 % and 9.68 %) were the main pathogens detected. The specific pathogen detection patterns across different age groups for in-patient and out-patient cases are shown in Figure 2 and Table 3.

**Table 3:** Detection of pathogens by age group among SARI cases.

Pathogen species	SARI				*P*
	0–4 years (*N* = 1,014) *n* (%)	5–14 years (*N* = 907) *n* (%)	15–59years (*N* = 739) *n* (%)	>60years (*N* = 1,695) *n* (%)	
SARS-CoV-2	44 (4.34%)	29 (3.20%)	60 (8.12%)	145 (8.55%)	**< 0.001**
IFV	82 (8.09%)	45 (4.96%)	59 (7.98%)	164 (9.68%)	**< 0.001**
RSV	203 (20.02%)	40 (4.41%)	6 (0.81%)	25 (1.47%)	**< 0.001**
HAdV	41 (4.04%)	62 (6.84%)	9 (1.22%)	14 (0.83%)	**< 0.001**
HMPV	56 (5.52%)	42 (4.63%)	13 (1.76%)	31 (1.83%)	**< 0.001**
HPIV	104 (10.26%)	45 (4.96%)	7 (0.95%)	34 (2.01%)	**< 0.001**
HCoV	28 (2.76%)	7 (0.77%)	8 (1.08%)	54 (3.19%)	**< 0.001**
HBoV	68 (6.71%)	17 (1.87%)	4 (0.54%)	12 (0.71%)	**< 0.001**
HRV	145 (14.30%)	124 (13.67%)	34 (4.60%)	52 (3.07%)	**< 0.001**
HEV	40 (3.94%)	30 (3.31%)	11 (1.49%)	15 (0.88%)	**< 0.001**
M.pneumoniae	148 (14.60%)	394 (43.44%)	49 (6.63%)	37 (2.18%)	**< 0.001**

Values in bold indicate statistically significant differences (*P* < 0.05).

### 3.3 Temporal distribution of respiratory pathogens

Monitoring results from Week 43 of 2023 to Week 38 of 2025 show that SARS-CoV-2 was detected continuously throughout the year, with the positivity rate exhibiting periodic fluctuations over time; the majority of epidemic peaks occurred between late winter and early spring, three distinct peaks in incidence were observed during the monitoring period, occurring in Week 11 of 2024, Week 35 of 2024 and Week 14 of 2025 respectively. Based on national SARS-CoV-2 surveillance data, multiple waves of minor COVID-19 outbreaks occurred across the country between 2024 and 2025. The peak period identified in this study for Hebei Province largely coincided with the period of widespread resurgence of COVID-19 nationwide, and the trends in the fluctuations of the outbreaks remained synchronized.

IFV exhibits a highly pronounced seasonal clustering pattern, characterized by concentrated outbreaks in winter and a significant reduction in prevalence during spring and summer. Two rounds of high-intensity outbreaks correspond precisely to the winter weeks of Weeks 3–7 of 2024 and Weeks 2–6 of 2025. Following the onset of spring, the positivity rate declined rapidly, remaining at extremely low levels throughout the vast majority of summer weeks, with only sporadic cases observed. Following the onset of spring, the positivity rate for influenza A virus declined rapidly; during the summer, the detection rate remained at an extremely low level for the vast majority of weeks, with an average detection rate of 0.32 %, and only sporadic cases were identified.

RSV circulation is concentrated in the winter and spring seasons, with extremely low positivity rates in summer; the peak of circulation consistently occurs during the transition from winter to spring, with the two peak periods falling in weeks 3–9 of 2024 and weeks 10–18 of 2025, respectively. HRV can be detected throughout the year, with no clear tendency for higher incidence in winter and spring; overall, the fluctuations in prevalence are moderate, with relatively higher levels of activity during the summer and autumn seasons.

HAdV incidence was concentrated in the early stages of surveillance, with a peak across the entire cycle occurring from week 47 of 2023 to week 7 of 2024, and several minor secondary peaks observed in weeks 15–31 of 2024; after week 35 of 2024, the overall positivity rate declined significantly, with only a slight increase observed in weeks 18–22 of 2025; the intensity of the epidemic has been decreasing year on year. HMPV exhibits an annual cyclical pattern, with two peaks occurring in week 47 of 2023 and from week 47 of 2024 to week 10 of 2025, respectively; detection levels remained low during the remaining periods. The peak of HPIV activity shifted later each year; early peaks were sporadic and relatively low, with the highest detection rate for the entire cycle occurring in week 26 of 2025. The peak season shifted from winter and spring to summer, with no fixed fluctuation cycle. M. pneumoniae generally exhibited high prevalence in the early stages and low prevalence in the later stages; Between Week 43 of 2023 and Week 47 of 2024, there were several spikes in prevalence, with the detection rate of M. pneumoniae reaching 15.27 % in the autumn of 2024. Following Week 48 of 2024, the positivity rate declined steadily, falling to 0.47 % by the autumn of 2025, with only sporadic, low-level positive cases reported.

**Figure 3:**
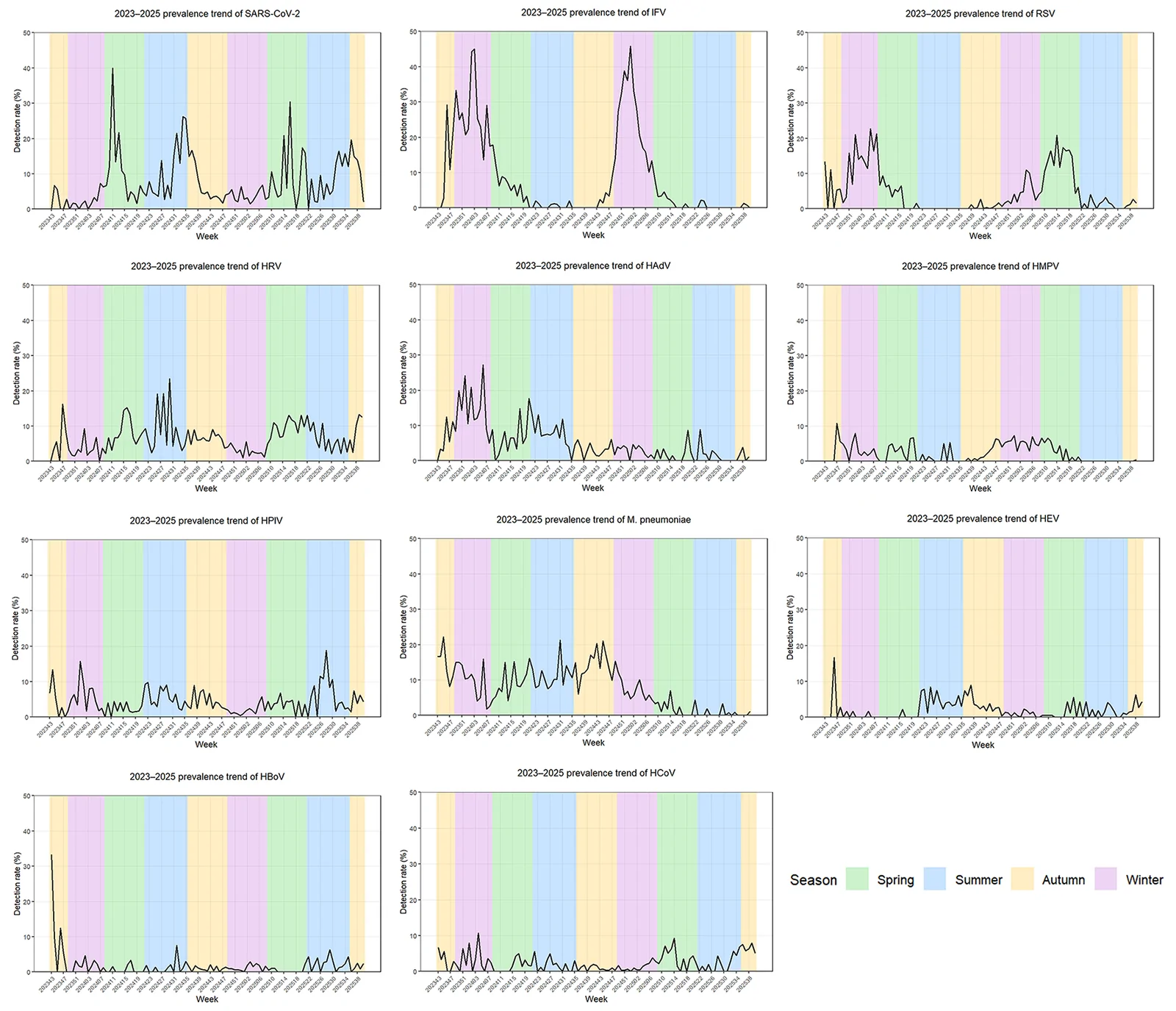
Charts showing the temporal trends of various pathogens in Hebei Province, 2023–2025; the horizontal axis represents the number of weeks in the year, and the vertical axis represents the pathogen detection rate

HEV peaked in week 47 of 2023, with several minor peaks occurring in mid-2024, followed by sporadic fluctuations at low levels in 2025; HBoV activity has weakened year on year, peaking at the end of 2023, with only sporadic minor fluctuations thereafter; HCoV showed steady fluctuations throughout the period with no severe outbreaks, and its relative peak was delayed until the first half of 2025. The temporal epidemiological trends of various pathogens in Figure 3.

### 3.4 Co-infection of pathogens

Among the 5,149 positive samples, there were 766 cases of mixed infection, representing a detection rate of 14.88 %; M. pneumoniae and HRV had the highest detection rate for mixed infections, with a total of 112 cases identified. Among the 1,991 hospitalized positive cases, a total of 473 cases of mixed infection were identified (23.76%), which was significantly higher than the number of mixed infections among positive cases in ILI (9.27%, 293/3,158). Among positive cases in ILI, HBoV accounted for the highest proportion of mixed infections (44.23%, 23/52), followed by HEV (39.84%, 49/123), HCoV (29.19%, 47/161) and HRV (27.59%, 109/395). Among positive in SARI cases, the highest proportion of co-infections was observed for HEV (77.08%, 74/96), followed by HBoV (70.30%, 71/101), HRV (59.15%, 210/355) and HAdV (56.35%, 71/126).

**Figure 4:**
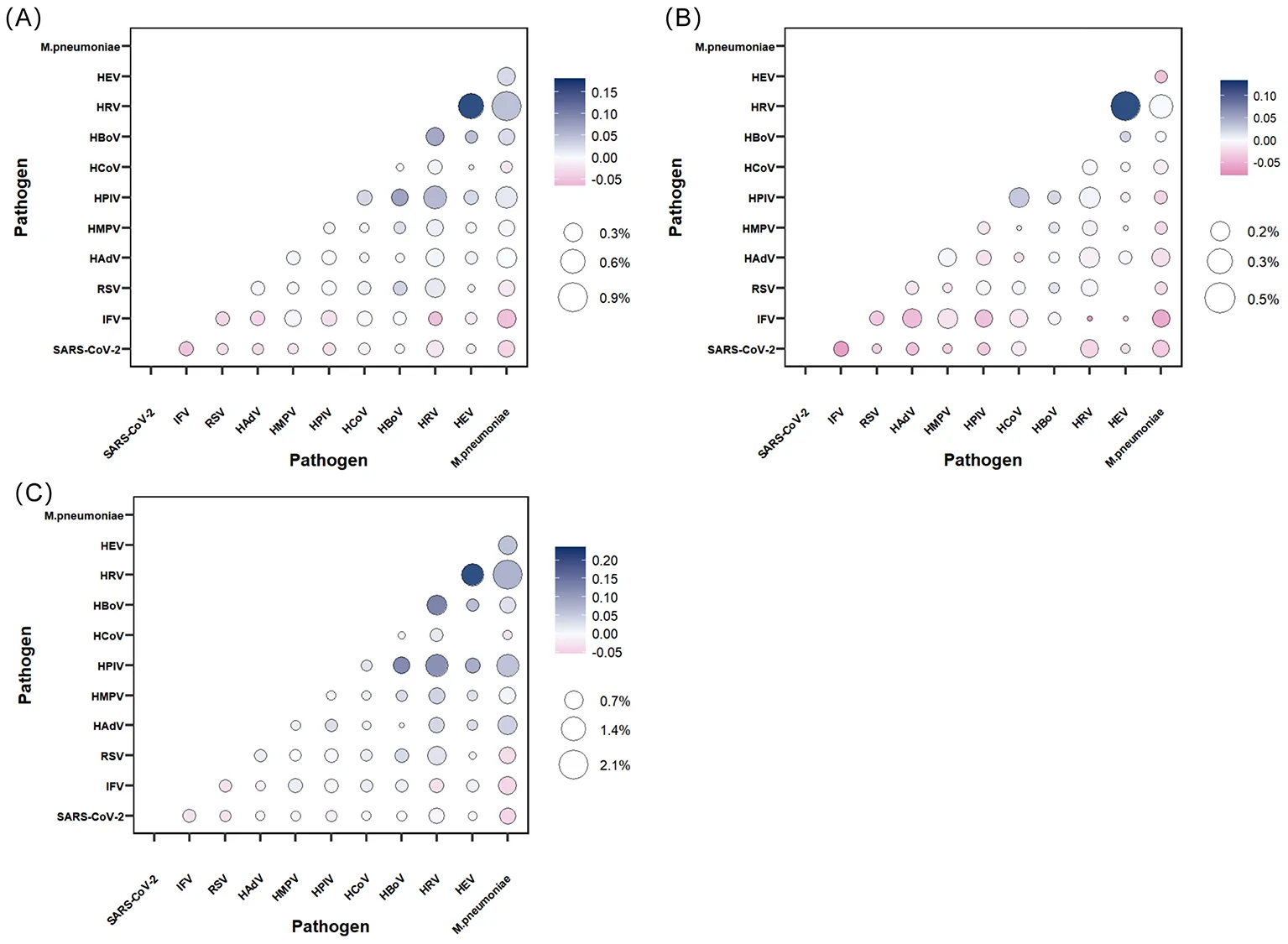
**A–C** illustrate the characteristics of co-infections with respiratory pathogens in the overall case cohort, the ILI case cohort and the SARI case cohort, respectively. In the figures, the size of the circles represents the frequency of co-detection of the two pathogens, whilst the shade of color indicates the magnitude of the Phi (ϕ) correlation coefficient.

Chi-square test results indicated statistically significant pairwise associations among the test results for various respiratory viruses. Among all pathogen pairs in ILI cases, the combination of HEV and HRV exhibited the statistically significant co-detection patterns (χ^2^ = 136.93, *P* < 0.001, φ = 0.134), suggesting that the simultaneous detection of these two viruses was more common in the study population. Furthermore, statistically significant co-detection patterns were also observed between HPIV and HCoV (χ^2^ = 10.534, *P* = 0.0012), HEV and HBoV (χ^2^ = 5.649, *P* = 0.017), and HPIV and HBoV (χ^2^ = 4.7423, *P* = 0.029). Compared with ILI cases, the statistical association coefficients for most pathogen pairs were generally higher among SARI, and there were also certain differences in the composition of pathogen combinations showing statistical association. Among the SARI population, the HEV–HRV pair remained the co-detection combination with the highest statistical association coefficient among all pathogen combinations (χ^2^ = 241.187, *P* < 0.001, φ = 0.235); In contrast to ILI, among SARI, statistically significant co-occurrence patterns were observed between HRV and HBoV (χ^2^ = 104.379, *P* < 0.001), between HPIV and HBoV (χ^2^ = 93.262, *P* < 0.001), and between HPIV and HRV (χ^2^ = 82.549, *P* < 0.001). The co-infection status and correlation of pathogens in ILI cases and SARI cases are shown in Figure 4.

## 4 Discussion

Acute respiratory infectious diseases are highly pathogenic and transmissible, posing a serious threat to human health; however, there are marked regional variations in their pathogen profiles and epidemiological characteristics (8, 9). In this study, the overall detection rate of respiratory pathogens in Hebei Province was 43.12%. A cross-regional comparison revealed that this detection rate was lower than the 58.04% recorded in north-western China (10) and the 65.7% recorded in Bulgaria (11); in Guangdong, southern China (12), the detection rate was 47.96 %, slightly higher than the results from this study in Hebei, whilst the positivity rate in this study was significantly higher than the 28.7% recorded in Chongqing (13). The variations in detection rates across different regions may be related to factors such as regional climate, lifestyle and the scope of pathogens tested for. In this study, over 56% of the samples tested negative for all 11 target pathogens. On the one hand, this study only tested for specified viruses and M. pneumoniae, and did not cover other atypical pathogens such as bacteria; consequently, infections caused by such pathogens could not be detected. On the other hand, retrospective sampling is difficult to standardize, and the substandard quality of some specimens may have led to false-negative results. Furthermore, in some patients, samples were collected too early or after they had entered the recovery phase, resulting in low pathogen loads. Combined with the fact that some individuals possessed strong immune responses, leading to rapid clearance of the pathogens, this ultimately resulted in negative test results.

The results of this study indicate that IFV, M. pneumoniae, SARS-CoV-2 and HRV are the primary pathogens causing respiratory infections in the Hebei region. Prior to the onset of the COVID-19 pandemic in 2019, surveillance of respiratory pathogens in Hebei and the North China region suggested that IFV, RSV and HRV were the predominant pathogens (14).

During the period of COVID-19 control measures, the circulation of droplet-transmitted pathogens in Hebei Province was significantly suppressed overall; the positivity rate for IFV remained at a low level for an extended period, the detection rate of HAdV was relatively low, and the usual seasonal pattern of RSV circulation was disrupted; HRV were less affected by epidemic control interventions and experienced a minor outbreak during the control period (15). Following the lifting of control measures in 2023, the detection rates of IFV, HAdV and RSV rebounded significantly, whilst M. pneumoniae exhibited a marked epidemic peak. This suggests that, following the end of control measures, the epidemiological intensity of most respiratory viruses rebounded; however, due to differences in transmission routes, the extent to which different pathogens were affected by epidemic prevention measures varied significantly.

It is worth noting that the pathogen detection rate in lower respiratory tract samples was higher than that in throat swabs, confirming that lower respiratory tract samples offer superior sensitivity for detecting respiratory pathogens. This imbalance in sample composition constitutes a significant confounding factor; the overall pathogen positivity rate for SARI was higher than that for ILI, a difference that cannot be entirely attributed to disease severity. The higher proportion of lower respiratory tract samples in the SARI group elevates the overall detection rate, thereby interfering with a direct comparison between the two groups. Due to clinical sampling principles, deep respiratory tract specimens are more readily obtained from critically ill patients. This study was unable to standardize the sampling types across all cases, making it difficult to fully correct for bias arising from differences in specimen type. Future prospective studies could standardize the sampling types to minimize the interference caused by specimen differences, thereby allowing the differences in pathogen prevalence between ILI and SARI to be more accurately attributed to disease severity.

In terms of age distribution, the findings of this study align with most domestic and international literature reports, indicating that the prevalence patterns of respiratory pathogens exhibit significant age specificity (16). Our results show that the highest proportion of pathogen infections occurs in the 5-14 age group, consistent with the conclusion from most studies that children are the primary susceptible population for respiratory infections (17). This is related to the living environment of school-age children and their still-developing immune systems. School-aged children spend extended periods in highly crowded collective settings, where frequent close contact significantly increases the risk and efficiency of respiratory pathogen transmission among student populations (18). Furthermore, the underdeveloped immune systems of children in this age group render them vulnerable to multiple pathogens (19). In summary, respiratory infections among school-aged children warrant heightened attention. Schools should strengthen health education and implement real-time monitoring when necessary. Relevant health authorities should also enhance group prevention and control measures for students while raising awareness of group prevention among both teachers and students.

From the age-specific distribution of pathogens, IFV was the most prevalent pathogen in both ILI cases across all age groups, a finding closely associated with its high transmissibility and widespread susceptibility among the population (20). SARS-CoV-2 detection rates were highest among individuals aged >60 years (8.21% in ILI cases settings, 8.55% in SARI cases), significantly exceeding other age groups. This aligns with findings from Kang SJ et al. (21) and may relate to immune decline, higher burden of underlying conditions, and differential exposure risks among the older adult(s). These results underscore that older adults remain a key priority group for SARS-CoV-2 infection prevention (22). Notably, all other pathogens primarily affected children aged 0–14 years: RSV, HRV, and HPIV were predominant in infants aged 0–4 years. This may stem from developmental characteristics of the pediatric immune system, where immaturity persists during infancy. Over time, maternal antibody levels decline, and infants exhibit insufficient endogenous IgA and IgM production (23, 24). Additionally, infants and young children have underdeveloped respiratory function and relatively narrow, fragile mucosal barriers, making them more susceptible to viral infections (25). The highest RSV detection rate was observed in hospitalized cases aged 0–4 years, suggesting a certain association between RSV infection and the occurrence of severe pediatric cases. For school-aged children aged 5–14 years, M. pneumoniae and HAdV were the predominant pathogens. This finding aligns with Chen B's report indicating that M. pneumoniae infection rates in children increase significantly with age (26). Comparing pathogen profiles between ILI cases and SARI cases revealed that the detection rate of M. pneumoniae (43.44%) was significantly higher in hospitalized children aged 5–14 years than in outpatient children (12.04%). There is a certain association between M. pneumoniae infection and severe cases requiring hospitalization (27).

The epidemiological trends of different pathogens vary markedly over time. IFV is most prevalent in autumn and winter, consistent with national surveillance data for the same period (28); this is likely closely related to the weakening of the human immune defenses as the weather turns colder and the increased risk of viral transmission due to greater indoor gatherings. In contrast to regions such as southern China, where temperatures remain moderate throughout the year, there is no significant cold winter to constrain viral activity (29). The SARS-CoV-2 virus exhibits two annual peaks in prevalence, concentrated in early autumn and early spring respectively; this pattern is also observed in common coronaviruses and is primarily driven by a combination of climatic factors, physiological changes and human behavior. Furthermore, the 6-month cycle of waning immunity within the population provides an opportunity for the virus to resurge (30, 31). HRV and HEV primarily exhibit a minor peak in prevalence during spring and summer, which is consistent with the findings of Lei Chengjiang et al. (10). HEV exhibits a marked preference for warm, humid conditions and demonstrates high environmental stability in such settings; furthermore, frequent person-to-person contact and exposure to water bodies during the summer facilitate its fecal-oral transmission (32); whereas the peak in HRV prevalence may stem from a ‘competitive release effect'—the presence of SARS-CoV-2 has a significant impact on HRV, with its positivity rate dropping markedly following the pandemic, suggesting that competition for shared host resources may exist between these two viruses (33). RSV is most prevalent during the winter and spring seasons, consistent with the findings of Ling Guo et al. (34). M. pneumoniae exhibits sporadic cases throughout the year, following a natural epidemic cycle of 3–7 years (35). In 2023, following the lifting of pandemic control measures, the proportion of MP-positive cases rose rapidly, consistent with reports from China indicating a widespread epidemic trend of M. pneumoniae in northern regions during the autumn of 2023 (36). In 2025, the detection rate of M. pneumoniae decreased markedly compared with 2023 and 2024. This may be attributed to an enhanced herd immunity barrier following the widespread epidemic of 2023–2024, resulting in a significant reduction in the regional susceptible population, as well as the strengthening of control measures.

Among the 5,149 positive samples, 766 cases involved mixed infections, indicating that a certain proportion of respiratory infection patients in this region experience mixed infections. The mixed infection rate was significantly higher among SARI cases than among ILI cases, suggesting that multiple pathogen infections may be a key factor contributing to increased disease severity and higher hospitalization risk. Regarding the tendency for mixed infections involving specific pathogens, the co-infection rates of HEV, HBoV, and HRV were particularly prominent among hospitalized pediatric patients. The most common co-infection combination was the simultaneous detection of M. pneumoniae and HRV in 112 cases, consistent with previous studies indicating that M. pneumoniae, as a primary pathogen, often acts synergistically with viruses to cause disease (37). Choo et al. (38) confirmed that HEV is the most common co-infecting virus in children with M. pneumoniae pneumonia. The high prevalence is attributed to the fact that M. pneumoniae and HRV are common respiratory pathogens in children, with overlapping seasonal patterns and susceptible populations. Furthermore, as the respiratory immune system in young children is not yet fully developed, viral co-infection exacerbates systemic inflammation and reduces the body's efficiency in clearing pathogens, ultimately increasing the detection rate of mixed infections. Statistical correlation analysis further revealed significant differences in pathogen co-infection patterns between outpatient and hospitalized populations: Among ILI cases, HEV and HRV exhibited the strongest association. Both belong to the Picornaviridae family and share a seasonal peak in spring and summer, suggesting that common environmental drivers or transmission routes may promote their co-epidemic circulation (39). In contrast, the pathogen association network among hospitalized patients was more complex. In addition to the persistent strong association between HEV and HRV, significant co-occurrence was observed between HRV and M. pneumoniae, as well as between HPIV and HRV, and between HPIV and HEV. Mixed infections occur more frequently among hospitalized patients, potentially due to the severity of mixed infections leading to hospitalization, increased cross-exposure risks in the hospital setting, and the use of more precise diagnostic methods for hospitalized patients compared to outpatient department patients. Furthermore, HBoV maintains a high mixed infection rate in both outpatient department and hospitalized cases, consistent with its characteristic role as a “bystander virus” frequently present in respiratory pathogen co-infections (40). It is worth noting that M. pneumoniae can be carried asymptomatically; a positive nucleic acid test result cannot definitively distinguish between a pathogenic infection and respiratory colonization. This issue is particularly pronounced in pediatric populations; therefore, the pathogenic role of Mycoplasma pneumoniae in mixed infections should be interpreted with caution. In summary, mixed infections are more likely to lead to severe illness and mortality. This highlights the need for clinicians treating severe respiratory infections to remain vigilant about the potential for multiple pathogens to act synergistically, avoiding the oversight of mixed infections' impact on disease progression by focusing solely on a single dominant pathogen.

This study has several significant methodological limitations. Firstly, the study applied different time requirements for sample collection from onset of illness for ILI and SARI cases: sampling for ILI cases was completed within 3 days of onset, whereas the sampling window for SARI cases could extend up to 10 days after onset. Previous studies on viral kinetics have shown that respiratory viral shedding typically peaks in the early stages of illness and gradually declines as the disease progresses. Consequently, the relatively longer sampling window for SARI cases makes it more likely that specimens from the middle to late stages of the disease will be included, which may systematically underestimate the viral positivity rate among SARI, resulting in a time-dependent detection bias. Secondly, the data in this study lack key confounding variables such as vaccination history, underlying comorbidities, pre-sampling antibiotic use, and socioeconomic status. The aforementioned factors can simultaneously influence both pathogen detection efficiency and disease severity; the absence of relevant data has prevented this study from conducting adequate analyses to adjust for confounding factors. Furthermore, this study did not explore the clinical significance of mixed infections involving multiple pathogens in depth. The study merely described differences in the detection rates of mixed infections among patients with SARI and ILI; due to limitations in the completeness of the retrospective medical records, it was not possible to systematically collect clinical indicators related to disease severity, such as the prevalence of pneumonia and length of hospital stay, making it difficult to conduct a quantitative analysis of the correlation between different combinations of co-infections and disease severity. To address these issues, future prospective studies should standardize the sampling time window for both ILI and SARI cases and systematically collect comprehensive information on vaccination history, underlying medical conditions, history of antibiotic exposure and socioeconomic indicators during patient enrolment. Third, this study conducted statistical analyses across multiple subgroups, covering age stratification, different pathogen types, and comparisons of co-infections involving multiple pathogens. Repeated hypothesis testing increases the probability of Type I errors (false positives), making it easy to misinterpret random fluctuations as statistical differences. Finally, the study did not undertake pathogen genotyping or testing for antimicrobial resistance; the lack of molecular-level data prevents an explanation of why local epidemiological patterns differ from those in other regions from the perspective of strain variation, further limiting the investigation into the underlying causes of regional epidemiological characteristics.

## 5 Conclusion

The overall pathogen positivity rate during the surveillance period was 43.12 %, with IFV, M. pneumoniae and HRV being the predominant pathogens. The overall positivity rate among inpatients (45.71 %) was significantly higher than that among ILI (41.62 %). The detection rate of M. pneumoniae among SARI aged 5–14 years was as high as 43.44%, suggesting that this age group is at high risk of M. pneumoniae infection and tends to present with more severe disease. Different pathogens circulated alternately, each exhibiting distinct seasonal patterns, such as the winter and spring peaks for IFV and RSV. The co-detection of pathogens (e.g. HRV and HBoV, HPIV and HBoV) was higher among SARI than among ILI, and the correlation coefficients were also higher. Based on the local epidemiological characteristics identified in this study, priorities for future public health efforts should be clearly defined. For example, given that infants and young children aged 0–4 years are susceptible to RSV and prone to developing severe respiratory infections, it is recommended that RSV be prioritized for screening in hospitalized infants and young children with respiratory infections, thereby enabling early identification and intervention to reduce the risk of severe respiratory infections in this age group; In view of the epidemiological pattern of high incidence of severe M. pneumoniae infections among school-aged children aged 5–14 years, it is recommended that routine Mycoplasma surveillance be implemented in schools, with screening of children presenting with fever and cough and tracking of absences, alongside strengthened early warning and prevention measures for cluster outbreaks during the start of the school term, thereby fortifying the defense against the disease within the school-aged population; Given the high detection rate and high co-infection rate of HRV in this region, as well as its prevalence in SARI, it is recommended that HRV be routinely included in the standardized pathogen diagnosis of severe pneumonia to avoid inadequate diagnosis and treatment resulting from missed co-infections. In summary, this study has clarified the epidemiological characteristics of respiratory infections in Hebei Province, including pathogen prevalence, population susceptibility, seasonal distribution and co-infection patterns. It provides robust data support and actionable practical guidance for tiered prevention and control of respiratory infectious diseases in the region, as well as for personalized, precision diagnosis and treatment and the optimisation of laboratory testing systems.

## Data Availability

The original contributions presented in the study are included in the article/Supplementary material, further inquiries can be directed to the corresponding authors.
